# What to consider when pseudohypoparathyroidism is ruled out: iPPSD and differential diagnosis

**DOI:** 10.1186/s12881-018-0530-z

**Published:** 2018-03-02

**Authors:** Arrate Pereda, Intza Garin, Spanish Network for Imprinting Disorders, E. Anda, S. Berrade, M. A. Ramos-Arroyo, R. Rodríguez Erdozain, A. Vicente, R. Rodriguez-Lopez, A. Moreno, M. Guitart, R. Oancea-Ionescu, O. Perez Rodriguez, S. Molinos Castro, E. Meriño, J. L. Salvador-Sanchis, P. López Mondejar, M. Zapico, E. Palomo, P. Rozas Moreno, F. Aleixandre-Blanquer, J. Argente Oliver, G. Martos, J. Pozo, O. Rubio-Cabezas, L. Bilbao Gasso, G. Marti, L. Martorell, R. Cardona, L. Suarez, S. Zambudio Sert, M. Obon, A. Sanchis Calvo, F. Moreno Macian, J. Cruz-Rojo, L. Garzon Lorenzo, J. Sanchez del Pozo, I. Riaño, M. Lahera Vargas, F. Blanco-Kelly, M. I. Lorda Sanchez, L. Soriano Guillen, S. Tahsin Swafiri, A. Azriel, B. Lecumberri, J. C. Moreno, V. Garcia Nieto, J. Garcia Diaz, R. Marin Iglesias, M. Martin Fuentes, A. Casteras, M. Clemente Leon, M. Ballesta-Martinez, M. J. Sanchez Soler, A. Gonzalez Meneses, J. Lopez Lopez, M. J. Garcia Barcina, B. Gener, I. Llano, M. Bonet Alcaina, Guiomar Perez de Nanclares

**Affiliations:** 1 Molecular (Epi)Genetics Laboratory, BioAraba National Health Institute, OSI Araba University Hospital, C/ Jose Atxotegi s/n, 01009 Vitoria-Gasteiz, Spain; 2 Department of Biochemistry and Molecular Biology, University of the Basque Country, Leioa, Spain

**Keywords:** Brachydactyly, Pseudohypoparathyroidism, Albright’s hereditary osteodystrophy, Hormone resistance, Short stature

## Abstract

**Background:**

Pseudohypoparathyroidism (PHP) is a rare disease whose phenotypic features are rather difficult to identify in some cases. Thus, although these patients may present with the Albright’s hereditary osteodystrophy (AHO) phenotype, which is characterized by small stature, obesity with a rounded face, subcutaneous ossifications, mental retardation and brachydactyly, its manifestations are somewhat variable. Indeed, some of them present with a complete phenotype, whereas others show only subtle manifestations. In addition, the features of the AHO phenotype are not specific to it and a similar phenotype is also commonly observed in other syndromes. Brachydactyly type E (BDE) is the most specific and objective feature of the AHO phenotype, and several genes have been associated with syndromic BDE in the past few years. Moreover, these syndromes have a skeletal and endocrinological phenotype that overlaps with AHO/PHP. In light of the above, we have developed an algorithm to aid in genetic testing of patients with clinical features of AHO but with no causative molecular defect at the *GNAS* locus. Starting with the feature of brachydactyly, this algorithm allows the differential diagnosis to be broadened and, with the addition of other clinical features, can guide genetic testing.

**Methods:**

We reviewed our series of patients (*n* = 23) with a clinical diagnosis of AHO and with brachydactyly type E or similar pattern, who were negative for *GNAS* anomalies, and classify them according to the diagnosis algorithm to finally propose and analyse the most probable gene(s) in each case.

**Results:**

A review of the clinical data for our series of patients, and subsequent analysis of the candidate gene(s), allowed detection of the underlying molecular defect in 12 out of 23 patients: five patients harboured a mutation in *PRKAR1A,* one in *PDE4D*, four in *TRPS1* and two in *PTHLH*.

**Conclusions:**

This study confirmed that the screening of other genes implicated in syndromes with BDE and AHO or a similar phenotype is very helpful for establishing a correct genetic diagnosis for those patients who have been misdiagnosed with “AHO-like phenotype” with an unknown genetic cause, and also for better describing the characteristic and differential features of these less common syndromes.

**Electronic supplementary material:**

The online version of this article (10.1186/s12881-018-0530-z) contains supplementary material, which is available to authorized users.

## Background

Albright’s hereditary osteodystrophy (AHO) is a unique phenotype classically associated with pseudohypoparathyroidism (PHP) [1, 2]. This phenotype was initially described by Albright et al. as a constellation of signs, including short stature, obesity with a rounded face, subcutaneous ossifications, mental retardation and brachydactyly. Parathyroid hormone (PTH) resistance was also originally included as a feature of the AHO phenotype as these authors noticed a reduced calcaemic and phosphaturic response to injected bovine parathyroid extract in such patients with normal renal function [1]. However, more patients with this phenotype but lacking hormone resistance were described in 1952, thus this disease was termed pseudopseudohypoparathyroidism (PPHP). Consequently, this type of hormonal resistance was included as a non-obligatory manifestation of AHO [3]. Many years later, genetic and/or epigenetic alterations at the guanine nucleotide-binding protein, alpha-stimulating (Gsα) locus (*GNAS*) were identified as the cause of this condition in about 70% of patients with a clinical diagnosis of PHP/PPHP [4], or iPPSD2 (inactivating PTH/PTHrP signalling disorder) and iPPSD3 according to the new proposed classification [5].

Despite this high detection rate of *GNAS* molecular defects, some patients with a clinical suspicion of PHP/PPHP still lack a confirmed molecular diagnosis, possibly due to the variability of the manifestations in terms of both number and severity, especially in cases in which there is no family history ([5–7] and personal data). In addition, the features of the AHO phenotype are not exclusive to PHP/PPHP. For example, AHO-like syndrome, or brachydactyly and mental retardation syndrome (BDMR, OMIM#600430), as its name indicates, includes a group of patients who show several features of AHO (BDE and mental retardation being the most notable) but with normal Gsα levels and with no endocrine abnormality [8]. These patients frequently carry deletions at the 2q37 chromosome or mutations in the gene coding for histone deacetylase 4 (*HDAC4*), which is found at this locus [8, 9]. Similarly, the biochemical alterations (hypocalcaemia and hyperphosphatemia) observed in PHP are also present in other syndromes associated with calcium homeostasis, such as hypoparathyroidism [10]. PTH resistance and brachydactyly (but in a more severe form) are also present in acrodysostosis with multihormonal resistance ACRDYS1 (or iPPSD4, OMIM#101800) [11–13], which is associated with mutations in the gene coding for the cAMP-dependent protein kinase type 1 regulatory subunit protein (*PRKAR1A*) [14]. Another type of acrodysostosis, which lacks hormone resistance (ACRDYS2 or iPPSD5, OMIM#6146139), is caused by mutations in the gene coding for phosphodiesterase 4D (*PDE4D*) [15, 16].

Considering recent publications in which a significant number of patients were clinically misdiagnosed as PHP when they actually had other syndromes [17], our goal was to validate our diagnostic algorithm starting with the brachydactyly feature to guide candidate gene testing in patients with features of AHO who do not carry genetic or epigenetic alterations at the *GNAS* locus.

## Methods

### Patients

The current series involved 23 out of a total of 149 patients referred to the Molecular (Epi)Genetics Laboratory at OSI Araba University Hospital for molecular diagnosis with a clinical suspicion of AHO phenotype with or without PTH resistance and the presence of brachydactyly type E or a similar pattern. (Epi)genetic alterations at the *GNAS* locus had been previously ruled out as described [18].

The clinical details of the whole series studied are summarized in Table 1. Some of these patients had already been reported, as indicated in the Table 1. The clinical history of the patients, including hand(s) (Additional files 1, 2 and 3: Figures S1-S3) and feet radiographs and clinical photos (if available), was requested from the physicians who referred the samples for genetic study.

**Table 1:** Clinical description of patients studied (patient data as provided by the clinician at the reference centre)

PATIENT	Age at consultation	Age of genetic diagnosis	Sex	Elevated PTH	Ca/P	Vitamin 25(OH)D	BD	MR	Height (cm)	BMI	Facial dimorphisms	Skeletal dysplasia	Advanced bone age	Dental defects	Other features
PHP01 (P9 [30])	7y	12y6m	M	Yes (and TSH)	N	ND	Severe and generalized	Behavioural disorder	−1,5SD	+ 1,3SD	broad face with widely spaced eyes	maxillonasal hypoplasia, severe hypoplasia of the skull, thickened calvarium, increased size of the jaw with severe malocclusion	No	ND	–
PHP02 (P8 [30])	6y6m	8y6m	F	Yes	P↑	ND	Severe and generalized	No	-2.5SD	0.2SD	broad face with widely spaced eyes,maxillonasal hypoplasia	severe hypoplasia of the skull, thickened calvarium	No	Yes	pigmented skin spots
PHP03 (P14 [30])	3y	3y10m	F	Yes (and TSH)	N	N	Severe and generalized	No	-1.8SD	0.9SD	broad face with widely spaced eyes	maxillonasal hypoplasia, severe hypoplasia of the skull, thickened calvarium, dysplasia of both hips	yes	ND	–
PHP04	13y	18y	F	Yes	N	ND	Severe and generalized (no X-ray)	No	135 cm (-3SD)	ND	Flat round face	genu valgum, Madelung deformity, exostosis in the knee (9y)	ND	ND	osteoporosis
PHP05	–	3y9m	F	Yes (and TSH)	ND	Low levels	Severe and generalized	ND	45 cm (−2.7 SD)	+ 1.8 SD	broad nasal root	–	Yes (5-6y)	ND	short neck, café-au-lait spots
PHP06 [31]	42y	45y	F	No (after Vit. D treatment) (and FSH	N	Low levels at first	Severe and generalized	severe	139 cm (− 4 SD)	>2SD	broad face with flattening of nasal ridge, facial dysostosis, spaced eyes	maxillonasal hypoplasia	–	ND	short neck, hyperinsulinism
PHP07 (P1 [33])	31y	40y	F	Yes (and low GH)	N	N	MT: III-V outcarving cones of MP & TP	learning difficulties (no test)	141.5 cm (−4 SD)	42.7 kg/m2 (> > 2SD)	round face, thin upper lip and prominent lower lip, pear-shaped nose	stubby fingers and toes	–	tooth hypoplasia	sparse hair, polyarthrosis, arthralgias of both hips and knees
PHP08 (P2 [33])	–	11y	F	N	N	N	MT: II-V outcarving cones of MP & BP	N	139.8 cm (-1SD)	25.6 kg/m2 (+ 1.96 SD)	long flat philtrum, and thin upper lip, pear-shaped nose, protruding ears	–	ND	ND	sparse hair, laterally sparse eyebrows, type 2 diabetes
PHP09	–	32y	F	No	N	ND	Generalized shortening, severe outcarving of the epiphyses	ND	152 cm (-2SD)	ND	thin upper lip, long philtrum, pear-shaped nose, sparse, eyebrows, prominent forehead	–	–	ND	Sparse hair
PHP09-D	5m	9m	F	No	N	ND	NA	ND	−2.8 SD	−2.4SD	thin upper lip, long philtrum, rounded nose, sparse eyebrows, prominent forehead, separated eyes	ND	ND	ND	Sparse hair
PHP10	–	33y	F	No	N	ND	Generalized shortening, stubby fingers (no X-ray received)	ND	ND	ND	thin upper lip, long philtrum, pear-shaped nose	ND	ND	ND	Sparse hair
PHP10-S		1y7m	M	No	N	ND	NA	ND	Growth failure	Growth failure	thin upper lip, long philtrum, rounded nose, sparse eyebrows, low-set ears	ND	ND	ND	Sparse hair, strabismus
PHP10-D		6y	F	No	N	ND	Yes (no X-ray)	ND	ND	ND	thin upper lip, long philtrum, rounded nose, sparse eyebrows, anteverted ears	ND	ND	ND	Sparse hair, strabismus
PHP11 (P3 [34])	11.5y	12y	F	N	N	N	MT: IV	N	−1 SD	N	No	–	Yes (13.5 years) (final height below target height)	No	Advanced bone age
PHP12 [35]	10y	12y	F	No	N	ND	MT: II-V TP: I & III	No	148.7 cm (−0.4SD)	1.7 SD	round face, long philtrum	–	Yes (12y)	No	short neck, descended and widely separated nipples
PHP13	28y	–	F	No	N	ND	Severe (especially IV & V MT) (no X-ray received)	Yes	139 cm (-4SD)	p3-p10	small saddle nose, prominent forehead, epicanthal folds, upward slanting palpebral fissures, low and dysplastic ears	maxillonasal hypoplasia with severe prognathism	No	micrognatia	fine and sparse hair, parse eyebrows, café-au-lait spots, severe myopia
PHP14	10y	–	F	No	N	ND	MT: V (no X-ray received)	ND	130.6 cm (-1SD)	85th	ND	clinodactyly, cone-shaped phalangeal epiphyses	ND	ND	–
PHP15	17y	–	F	No	N	ND	MT: IV (no X-ray received)	No	p45	p75-p90	round face, facial asymmetry	–	ND	ND	hypothyroidism
PHP16	16y	–	M	No (after Vit. D treatment)	N (after Vit. D treatment)	Low levels at first	MT: IV & V (no X-ray received)	ND	ND	(Obesity)	–	–	–	Dental malformations	acanthosis nigricans, short neck, hyperinsulinism
PHP17	9y7m	–	F	No	N	ND	MP: II-V at least. (BDA1?)	ND	−2,2SD	+ 2,7SD	prominent forehead, depressed nasal root	rhizomelia	No	ND	increased subarachnoid space, ventriculomegaly, bilateral frontotemporal cortical atrophy, ectopic neurohypophysis
PHP18	9y11m	–	M	No	N	N	Stubby digits MT: III-IV at least	No	117 cm (−3,38SD)	N	flattening of nasal ridge	stocky build, hip hypoplasia, horizontal acetabulum, varum deformity, shortened tibia and femur, decreased interpedicular distance, scoliosis, bone dysplasias	ND	ND	–
PHP19	65y	–	F	No	N	N	MT: IV	No	(SS)	ND	big nose, thin upper lip	–	–	ND	–
PHP20	15y5m	–	M	No	N	N	MT: IV & V	No	143,5 cm (−3,9SD)	+ 4,22SD	ND	–	ND	ND	delayed puberty
PHP21	10y	–	F	N (TSH mildly increased)	N	ND	MT: IV; TP: I	No	p30	N	prominent forehead, periorbital hyperpigmentation, long palpebral fissure, deep philtrum, thick eyebrows	–	ND	ND	–
PHP22	13y	–	F	N	N	ND	MP: II & V (BDA4?)	No	p50	+ 1.5SD	N	Bilateral cubitus valgus, short forearms, exostosis in both tibia, dorsolumbar hyperkyphosis in D12-L1	ND	N	bicornuate uterus, short neck, wide thorax
PHP23	8y1m	–	F	No	N	N	MT: IV & V (mild) and clinodactyly of V	Mild	+3SD	> + 3SD	round face, thin upper lip, pear-shaped nose, sparse, arched eyebrows	–	ND	ND	sparse hair, epilepsy

*P* patient, *S* son, *D* daughter, *PTHr* PTH resistance, *Vit. D* vitamin D, *Ca* calcemia, *P* phosphatemia, *BD* brachydactyly, *MR* mental retardation, *N* normal, *NA* not assessable due to short age, *ND* no data, *MT* metacarpal, *TP* telophalanx, *MP* mesophalanx, *BP* basophalanx, *X-ray* radiography, *SD* standard deviation, *SS* short stature

### Candidate gene approach

**Fig. 1:**
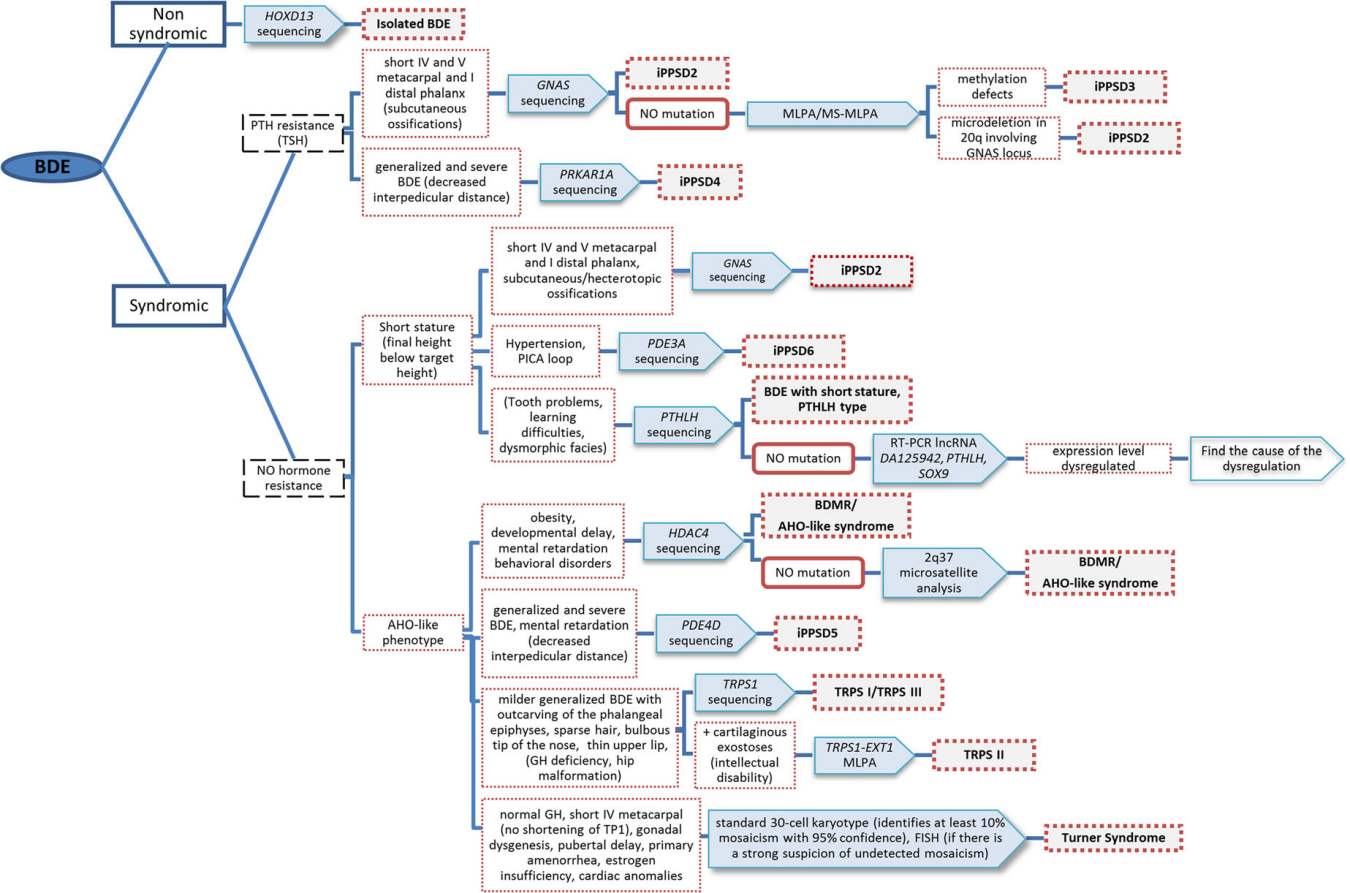
Updated clinical algorithm used during this project. Features in parentheses indicate that they are not obligatory features of the syndromes. BDE: brachydactyly type E; MLPA: multiplex ligation-dependent probe amplification; MS-MLPA: methylation specific-MLPA; iPPSD: inactivating PTH/PTHrP Signaling Disorder

The patients’ clinical features were reviewed to classify them according to the brachydactyly pattern and other clinical features, in accordance with the diagnostic algorithm proposed by us previously [19] and updated to include the most recent findings (Figure 1). The most probable candidate gene(s) were studied in each patient (Additional file 4: Table S1).

The following disorders (all of which present BDE or similar types) were considered: (i) iPPSD4 [14] and iPPSD5 [15, 16]; (ii) hypertension with brachydactyly syndrome or iPPSD6 (HTNB, OMIM#112410), in which the responsible gene, phosphodiesterase 3A (*PDE3A*), has been identified very recently [20]; (iii) tricho-rhino-phalangeal syndrome type I and III (TRPS-I, OMIM#190350; TRPS-III, OMIM#190351), caused by mutations in the *TRPS1* gene [21] or the more severe form, type II (OMIM#150230), which is a contiguous gene syndrome on 8q24.1 involving loss-of-function copies of the *TRPS1* and *EXT1* genes [22]; (iv) BDMR; (v) brachydactyly type E with short stature, *PTHLH* type (OMIM#613382), caused by mutations in the gene coding for parathyroid hormone-related protein (*PTHLH*) [23–25]; and (vi) isolated BDE in which the *HOXD13* gene has been implicated [26, 27].

We should mention at this point that, although Turner syndrome chromosomal disorder (frequently, 45,X) is a relatively well-known entity, patients also show BDE [28] and short stature, which could give rise to some misdiagnoses. However, none of our patients presented clinical features compatible with Turner syndrome.

### Mutational analysis of candidate genes

Genomic DNA was extracted from peripheral blood mononuclear cells using the QIAamp DNA Mini Kit (QIAGEN, Düren, Germany) according to the manufacturer’s instructions.

The DNA obtained was amplified by PCR for *PRKAR1A* (ref: NM_002734), *PDE4D* (ref: NM_001104631), *TRPS1* (ref: NM_014112), *HDAC4* (ref: NM_006037), *PTHLH* (ref: NM_198965.1) and/or *HOXD13* (ref: NM_000523) coding exons and exon–intron junction, using specific primers (primers available on request). Direct Sanger sequencing was carried out using standard methods and an ABI 3500 Genetic Analyzer (Applied Biosystems, Foster City, CA). The reference sequences mentioned in parentheses were employed for mutation description according to the HGVS nomenclature.

### Gene dosage analyses by multiplex ligation-dependent probe amplification (MLPA)

Gene dosage analyses were carried out using the SALSA MLPA P228-B1 TRPS1-EXT1, P179-B1 Limb− 1 and P264 B1 Human Telomere 9 probemix (MRC-Holland, Amsterdam, The Netherlands), when no alterations were identified by direct sequencing of the *TRPS1* gene, *HOXD13* gene or *HDAC4* gene, respectively.

### Microsatellite analysis

2q37 deletions were studied by microsatellite analysis as reported previously [29].

## Results

Following the proposed candidate gene approach guided by the aforementioned diagnostic algorithm, 12 out of 23 patients were diagnosed genetically.

### Mutations in *PRKAR1A* in the current series: iPPSD4

Six patients with PTH resistance and severe, generalized brachydactyly, both of which are typical characteristics of iPPSD4 (classically called ACRDYS1), were identified. Accordingly, the *PRKAR1A* gene was sequenced. Four of these patients harboured the recurrent c.1101C>T/p.Arg368* mutation (PHP01 and PHP02 already reported [30], and PHP04 and PHP05) and a fifth one carried the c.854A>G/p.Gln285Arg mutation (PHP03, reported [30]). The functional impact of this substitution was assessed experimentally and it was found to produce a similar impairment to the recurrent mutation, i.e., defects in PKA activation characterized by a reduced sensitivity to cAMP [30]. Parental studies suggested that all mutations were *de novo*. The remaining patient had no mutation in *PRKAR1A*.

### Mutations in *PDE4D* in the current series: iPPSD5

In the patient (PHP06) with no mutation in *PRKAR1A*, the other gene, *PDE4D*, associated with iPPSD5 (formerly named ACRDYS2) was sequenced. Initially this patient presented elevated PTH (PTH = 107 pg/ml, normal range: 10–65; 25-OH vitamin D = 13.9 ng/ml, normal range 20–100), which is why iPPSD4 was suspected. However, after vitamin D treatment her PTH level normalized [31] (it was probably secondary to vitamin D deficiency) [32]. A novel heterozygous mutation (c.934C>G/p.Leu312Val) was identified in *PDE4D*. Functional studies of this mutation confirmed its pathogenicity [31].

### Mutations in *TRPS1* in the current series: TRPS-I

Four patients with features suggestive of TRPS-I (i.e. short stature, severe BDE with outcarving of the phalangeal epiphyses, sparse hair, bulbous tip of the nose or pear shaped nose, long philtrum, thin upper lip, as described in table 1) were positive for *TRPS1* gene mutation: two patients (PHP08, previously described [33]; and PHP09) carried the recurrent c.2762G >C/p.Arg921Gln mutation and the remaining two patients each showed a different mutation: c.2830delA/p.Arg944Glyfs*3 in one case (PHP07, previously described [33]) and c.3159_3160delAAinsT/p.Lys1053Asnfs* (PHP10) in the other. Although neither of these has been described previously in the literature, the cosegregation in other family members (Additional file 4: Table S1) and frameshift characteristics (i.e., they would lead to truncated proteins if translated) suggested that they were possibly pathogenic.

### Mutations in *PTHLH* in the current series: BDE with short stature *PTHLH* type

The BDE *PTHLH* type was suspected in two patients with BDE and advanced bone maturation for their chronological age. Indeed, two different novel mutations (c.101+ 3delAAGT, in PHP11 [34] and c.166C>T/p.Arg56*, in PHP12 [35]) were identified in the *PTHLH* gene in these patients. The characteristics of these mutations (frameshift and nonsense, respectively) suggested that they were causative of the pathology.

## Discussion

PHP includes a heterogenic group of rare disorders associated with the AHO phenotype [1, 2]. Except for subcutaneous ossifications, the features of AHO are rather nonspecific as they also appear in other disorders, such as AHO-like syndrome [8] or acrodysostosis [11–13]. Less frequently, misdiagnosis with other entities has been observed because of the presence of BDE combined with short stature and obesity, which are also typically associated with other dysmorphic features and sometimes also with hormonal imbalances [33–37]. In this constellation of features, obesity or overweight and short stature could act as confusing factors as both are nonspecific [5, 33]. In addition, although obesity, intellectual disability, and resistance to several hormones are still extensively related to AHO, they may not be directly associated with genetic defects in *GNAS* [5].

The discovery of new genes implicated in syndromes with a phenotype similar to AHO, as well as other molecular mechanisms causative of iPPSD2 (classically named PHP/PPHP) [38], has been very helpful for establishing a correct genetic diagnosis for patients diagnosed with an “AHO-like phenotype” of unknown genetic cause [11, 14, 17, 33–35, 39], as well as for better describing the characteristic features of these less common syndromes. In ours and previously reported experiences [19, 40], BDE is the most specific and objective feature. For this reason, it was used as the inclusion criterion in this study and as a starting point to classify the aforementioned disorders in the previously proposed diagnostic algorithm [19]. It is well known that BDE was initially described as a variable shortening of the metacarpals/metatarsals with a more or less normal length of the phalanges [41].

As a result of the clinical re-evaluation of this series of patients, half of them (12/23) could be genetically diagnosed (supporting the importance of a good clinical examination, and the need of multidisciplinary approaches in the follow-up of these patients) and new knowledge acquired regarding these pathologies and the characteristic features detailed below.

We also analysed the features observed in our iPPSD4 (5/12) and iPPSD5 (1/12) patients and other cases described in the literature and noticed that the skeletal dysmorphisms (broad face, widely spaced eyes, maxillonasal hypoplasia, severe and generalized brachydactyly in hands/feet, severe short stature, cone-shaped epiphyses with early epiphyseal fusion, and advanced bone age [17, 30]) are very similar in both groups, although the facial dysmorphisms are often more severe in iPPSD5 [17, 30, 42]. Decreased interpedicular distance and mental retardation also appear to be more specific for iPPSD5 [6, 17, 30] since iPPSD4 patients show only behavioural disorders [30]. Finally, hormone resistance, which was initially used as a main differential characteristic to classify the patients with acrodysostosis, seems not to be as specific as initially appeared because more exceptions are found as more patients are reported (PTH resistance was recorded in 76% of iPPSD4 and 27% of iPPSD5 cases in the last review of Elli et al. [17]). All the iPPSD4 patients in our series exhibited PTH resistance, and although the iPPSD5 patient (PHP06) initially presented elevated PTH levels, PTH normalized after correcting the vitamin D deficiency, which is consistent with secondary hyperparathyroidism [32]. It is noteworthy that in contrast with the rest of the syndromes reflected in the algorithm, in which brachydactyly is usually not marked until the age of 6 years [39, 40], in acrodysostosis (both iPPSD4 and iPPSD5) the shortening and cone-shaped epiphyses are manifested during early childhood [16, 30].

Given the presence of brachydactyly and short stature, TRPS could be confused with the AHO phenotype, especially so if obesity (or overweight) and/or PTH resistance [37] and another hormone imbalance (GH deficiency has also been reported in some TRPS cases [43–47]) appear in the clinical profile, as shown in our two previously published cases [33] reviewed here. Keeping in mind all the identified cases in our series of patients, and comparing with those reported previously, in our opinion the most characteristic and illustrative features of TRPS syndrome are: bulbous tip of the nose (or pear-shaped nose), thin upper lip, involvement of the phalanges in the brachydactyly pattern and the typical outcarving of the phalangeal epiphyses, and sparse, slowly growing scalp hair.

Alterations which lead to the haploinsufficiency in *PTHLH*, the gene coding for parathyroid hormone related protein (PTHrP), have been identified as a cause of autosomal-dominant BDE in 11 families, two of them within our series [23, 24, 34, 35, 48, 49]. Although initially named as “BDE with short stature, *PTHLH* type” (OMIM#613382), because it is almost always associated with short stature [23–25], we have observed in both *PTHLH* patients in our series (PHP11 [34] and PHP12 [35]) that this short stature may not manifest until middle or late childhood. In both these cases, the patients had normal stature for their age but advanced bone age. Consequently, they experienced early epiphyseal closure, an early halt to growth, and their predicted final height is estimated to be below their target height. Thus, both the progenitors’ final height and bone age should be taken into account when determining whether patients show a height in the lower range of normality.

Overall, our use of a diagnostic algorithm in the current study has helped to determine the genetic cause in 12/23 patients with BDE who were clinically misdiagnosed as PHP/PPHP. Similarly to the 12 cases solved, the remaining cases were also classified and studied using the candidate gene approach guided by the proposed algorithm. However, we did not find any genetic alterations in the candidate genes studied, possibly due to some limitations of the study, such as (i) analysis of putative deletions at *PTHLH* is lacking; (ii) hand X-rays are missing for four patients, therefore it is difficult to propose any other potential diagnosis, (iii) although a large number of genes have been identified as the cause of BDE in recent years, the genetic cause of some BDE cases remains unknown [50].

## Conclusions

We conclude that use of the presented algorithm in patients with idiopathic BDE is helpful for establishing a correct genetic diagnosis for those patients who have been misdiagnosed as PHP/PPHP. [5]

## Additional files

Additional file 1:Figure S1. **Figure S1.** Hand X-rays for patients with acrodysostosis, caused by mutation at either *PRKAR1A* (PHP02, panel **A**) or *PDE4D* (PHP06, panel **B**) They presented severe shortening of all hand bones with cone-shaped epiphysis (rows). (TIFF 1641 kb)

Additional file 2: **Figure S2.** Hand X-rays for a mother (PHP09, panel **A**) and her daughter (PHP09-D, panel **B**) with tricho-rhino-phalangeal syndrome caused by the same mutation in *TRPS1*. The mother’s hands showed severe bilateral shortening of the bone with the characteristic outcarving of the phalangeal epiphysis (row). However her daughter was too young to manifest this brachydactyly and outcarving. (TIFF 3376 kb)

Additional file 3: **Figure S3.** Hand X-rays for patients without genetic diagnosis: **(A)** Patient PHP18 exhibits stubby digits and shortening of at least metacarpals (MT) III-IV; **(B)** Patient PHP19’s hands show bilateral shortening of MT IV; **(C)** Patient PHP20 presents shortening of MT IV and V; **(D)** Patient PHP21’s hand reveals bilateral shortening of MT IV and first telophalanx; **(E)** Patient PHP22’s hands present bilateral shortening of II and V mesophalanges (similar to BDA4); **(F)** Patient PHP23 presents mild shortening of MT IV and V and clinodactyly of the V digit. (TIFF 3217 kb)

Additional file 4: **Table S1.** Brief summary of the candidate genes analysed for each patient and the results. (DOCX 21 kb)

## Abbreviations

ACRDYS1: Acrodysostosis type 1with multihormonal resistance
ACRDYS2: Acrodysostosis type 2 without hormone resistance
AHO: Albright’s hereditary osteodystrophy
BDE: Brachydactyly type E
BDMR: Brachydactyly and mental retardation syndrome
GH: Growth hormone
*GNAS*: Gene coding alpha subunit of the stimulatory guanine nucleotide-binding protein
Gsα: Gs protein alpha subunit
*HDAC4*: Gene coding for histone deacetylase 4
*HOXD13*: Gene coding for homeobox D13
HTNB: Hypertension with brachydactyly syndrome
iPPSD: inactivating PTH/PTHrP signalling disorder
*PDE3A*: Gene coding for phosphodiesterase 3A
*PDE4D*: Gene coding for phosphodiesterase 4D
PHP: Pseudohypoparathyroidism
PKA: Protein kinase type 1A
PPHP: Pseudopseudohypoparathyroidism
*PRKAR1A*: Gene coding for the cAMP-dependent protein kinase type 1 regulatory subunit
PTH: Parathyroid hormone
*PTHLH*: Gene coding for parathyroid hormone-related protein
PTHrP: Parathyroid hormone related protein
TRPS: Tricho-rhino-phalangeal syndrome
*TRPS1*: Gene coding for zinc finger transcription factor TRPS1
