# Supplementary figures and images for: What to consider when pseudohypoparathyroidism is ruled out: iPPSD and differential diagnosis
Source: BMC Med Genet. 2018 Mar 2;19:32. doi: 10.1186/s12881-018-0530-z (PMC5834905; doi:10.1186/s12881-018-0530-z)

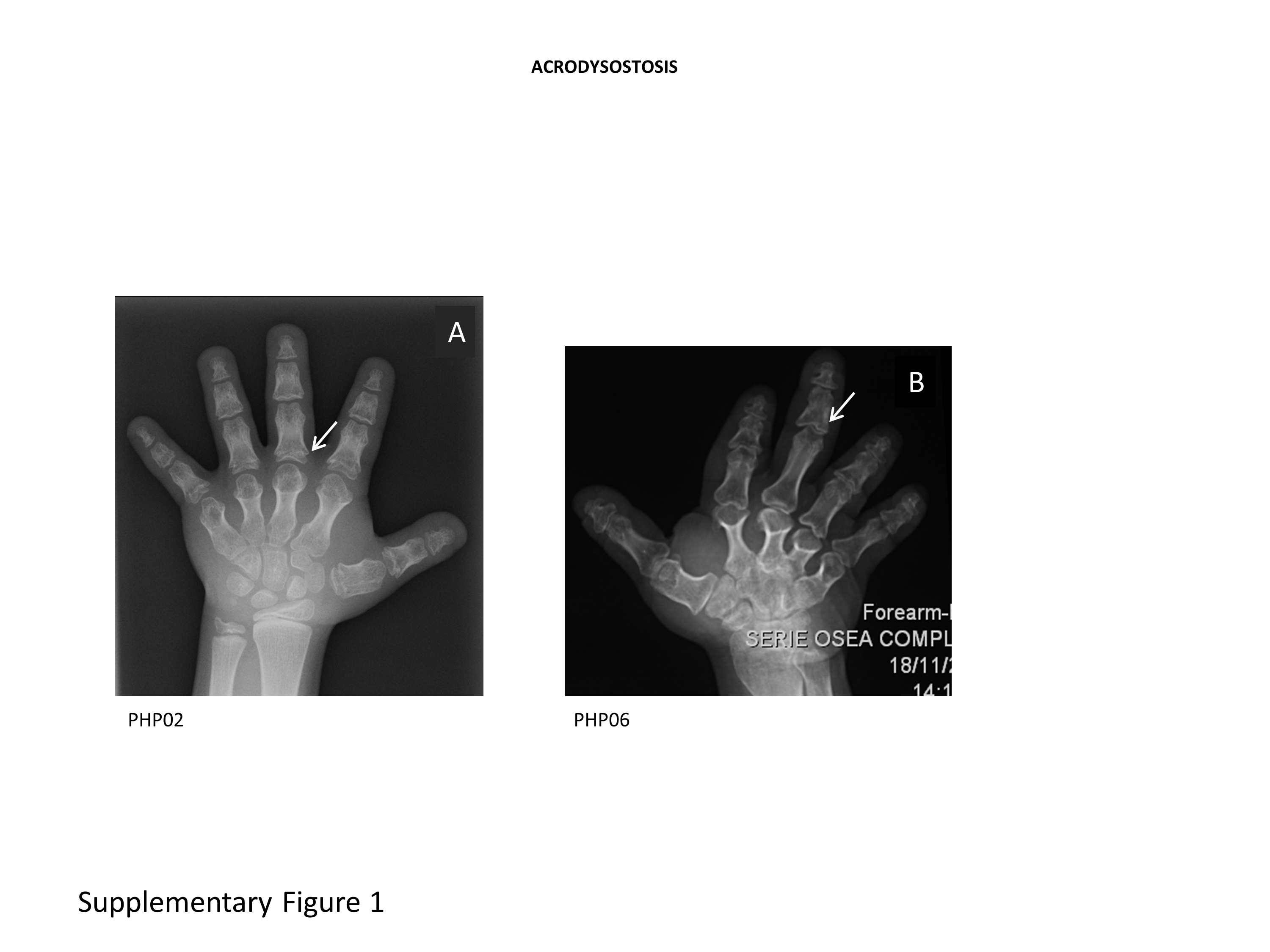

Supplement: Supplementary file 1 — Figure S1. Hand X-rays for patients with acrodysostosis, caused by mutation at either PRKAR1A (PHP02, panel A) or PDE4D (PHP06, panel B) They presented severe shortening of all hand bones with cone-shaped epiphysis (rows). (TIFF 1641 kb) [file 12881_2018_530_MOESM1_ESM.tif]

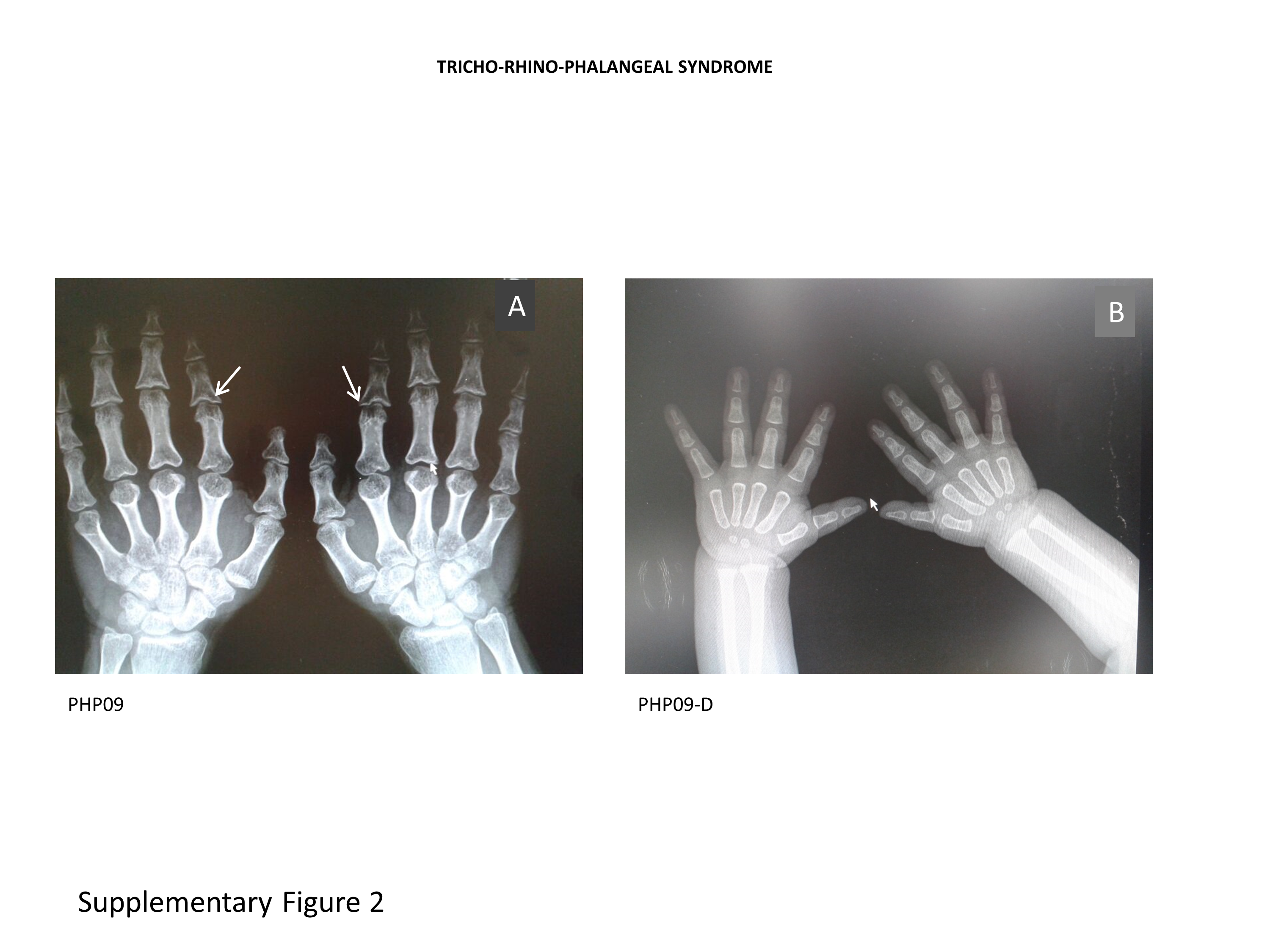

Supplement: Supplementary file 2 — Figure S2. Hand X-rays for a mother (PHP09, panel A) and her daughter (PHP09-D, panel B) with tricho-rhino-phalangeal syndrome caused by the same mutation in TRPS1. The mother’s hands showed severe bilateral shortening of the bone with the characteristic outcarving of the phalangeal epiphysis (row). However her daughter was too young to manifest this brachydactyly and outcarving. (TIFF 3376 kb) [file 12881_2018_530_MOESM2_ESM.tif]

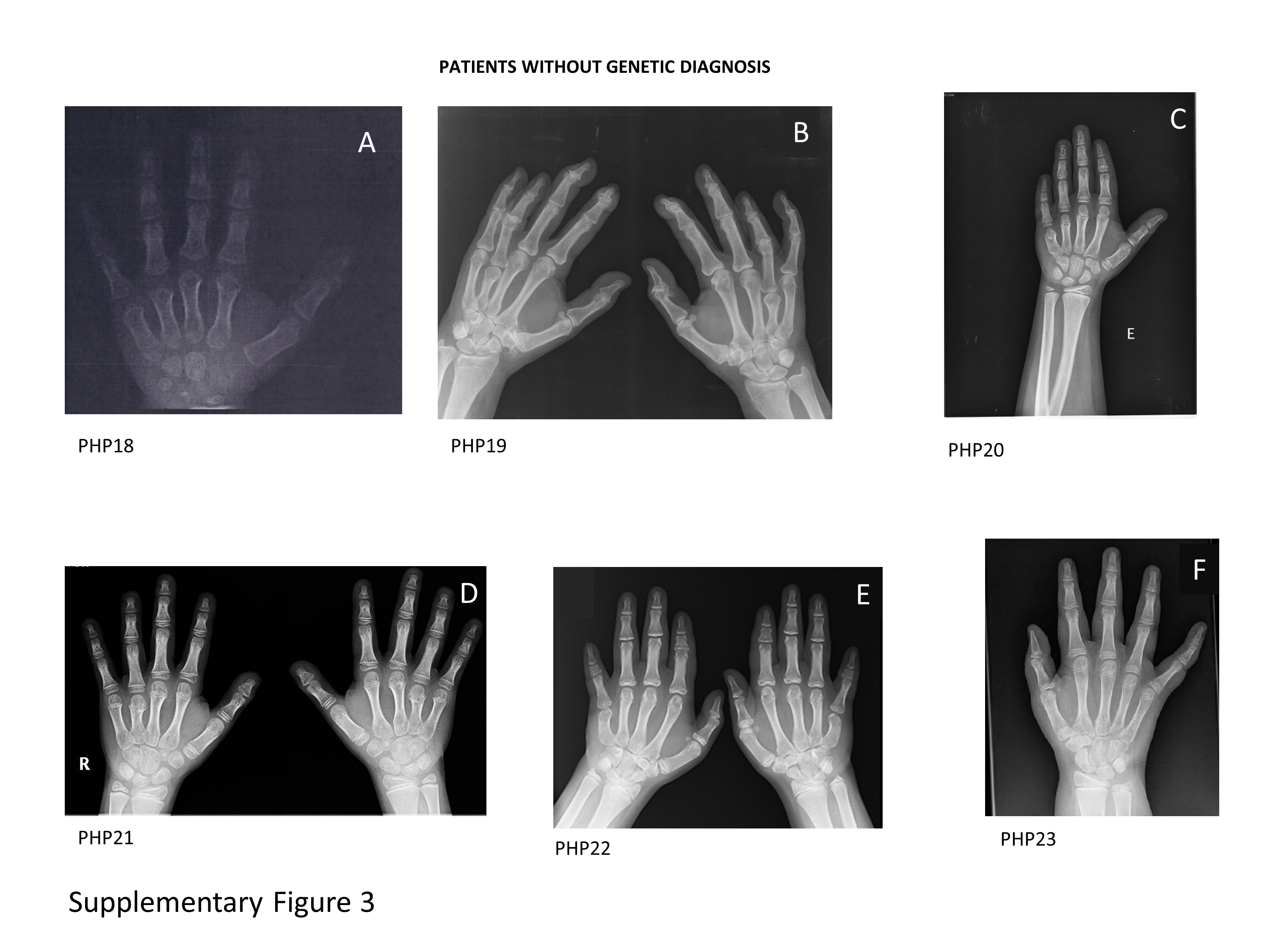

Supplement: Supplementary file 3 — Figure S3. Hand X-rays for patients without genetic diagnosis: (A) Patient PHP18 exhibits stubby digits and shortening of at least metacarpals (MT) III-IV; (B) Patient PHP19’s hands show bilateral shortening of MT IV; (C) Patient PHP20 presents shortening of MT IV and V; (D) Patient PHP21’s hand reveals bilateral shortening of MT IV and first telophalanx; (E) Patient PHP22’s hands present bilateral shortening of II and V mesophalanges (similar to BDA4); (F) Patient PHP23 presents mild shortening of MT IV and V and clinodactyly of the V digit. (TIFF 3217 kb) [file 12881_2018_530_MOESM3_ESM.tif]
