# Supplementary material for: What to consider when pseudohypoparathyroidism is ruled out: iPPSD and differential diagnosis
Source: BMC Med Genet. 2018 Mar 2;19:32. doi: 10.1186/s12881-018-0530-z (PMC5834905; doi:10.1186/s12881-018-0530-z)
Supplement: Supplementary file 4 — Table S1. Brief summary of the candidate genes analysed for each patient and the results. (DOCX 21 kb) [file 12881_2018_530_MOESM4_ESM.docx]

**Additional file 4: Table S1.** Brief summary of the **c**andidate genes analyzed for each patient and the results.

| **PATIENT** | **MOLECULAR STUDIES** | | | | | | | **Inheritance** |
| --- | --- | --- | --- | --- | --- | --- | --- | --- |
|  | ***PRKAR1A*** | ***PDE4D*** | ***PTHLH*** | ***HOXD13*** | ***TRPS1*** | ***HDAC4*** | **2q37**  **microsatellites** |  |
| **PHP01**  **(P9 [30])** | c.1101C>T/  p.Arg368* | - | - | - | - | - | - | *De novo* |
| **PHP02**  **(P8 [30])** | c.1101C>T/  p.Arg368* | - | - | - | - | - | - | *De novo* |
| **PHP03**  **(P14 [30])** | c.845A>G /  p.Gln285Arg | - | - | - | - | - | - | *De novo* |
| **PHP04** | c.1101C>T/  p.Arg368* | - | - | - | - | - | - | *De novo* |
| **PHP05** | c.1101C>T/  p.Arg368* | - | - | - | - | - | - | *De novo* |
| **PHP06**  **[31]** | N | c.934C>G/  p.Leu312Val | - | - | - | - | - | n. a.* |
| **PHP07**  **(P1 [33])** | - | - | - | - | c.2830delA/  p.Arg944Glyfs*3 | - | - | Fam.  (transmitted to her daughter [33]) |
| **PHP08**  **(P2 [33])** | - | - | - | - | c.2762G>A/  p.Arg921Gln | - | - | *De novo* |
| **PHP09** | - | - | - | - | c.2762G>A/  p.Arg921Gln | - | - | Fam. (transmitted to her daughter, Table 1) |
| **PHP10** | - | - | - | - | c.3159_3160delAAinsT/  p.Lys1053Asnfs* | - | - | n. a.** (transmitted to 2 children, Table 1) |
| **PHP11**  **(P3 [34])** | - | - | c.101+3delAAGT | - | - | - | - | *De novo* |
| **PHP12**  **[35]** | - | - | c.166C>T/p.Arg56* | - | - | - | - | Pat (healthy, mosaic) |
| **PHP13** | N | N | - | - | N | N | N | - |
| **PHP14** | - | - | N | N | N | - | - | - |
| **PHP15** | - | - | N | N | - | - | - | - |
| **PHP16** | - | - | N | N | - | N | - | - |
| **PHP17** | - | N | N | - | - | N | N | - |
| **PHP18** | - | N | N | - | - | - | - | - |
| **PHP19** | - | - | N | N | N | - | - | - |
| **PHP20** | - | - | N | N | - | N | - | - |
| **PHP21** | - | - | N | N | - | - | - | - |
| **PHP22** | - | - | N | N | - | - | - | - |
| **PHP23** | - | - | N | N | N | N | - | - |

N: normal; -: not analyzed; fam.: familial (siblings affected, no parent testing); mat: maternally inherited; pat: paternally inherited; *: lack of paternal sample; **: lack of parental sample.
